# Global Goals and Global Sustainability

**DOI:** 10.3390/ijerph13100991

**Published:** 2016-10-07

**Authors:** Subhash Janardhan Bhore

**Affiliations:** Department of Biotechnology, Faculty of Applied Sciences, AIMST University, Bedong-Semeling Road, 08100 Bedong, Kedah, Malaysia; subhash@aimst.edu.my or subhashbhore@gmail.com; Tel.: +60-4-429-8176

**Keywords:** global sustainability, global goals, go green, poverty, pollution, public health, sustainability

On 25 September 2015, the United Nations (UN) member countries adopted an ambitious 17 Sustainable Development Goals (SDGs) aiming to ‘transform the world’ in the next 15 years. The implementation of the plan began in January 2016. The ultimate goal of the plan is to promote ‘public health and development’ and ‘global sustainability’ [1]. If everything goes smoothly, we will be able to see a reasonably better world by the end of 2030 and one can expect ‘zero poverty’, ‘no shortage of food’, and ‘healthy populations’ along with alleviated societal problems [2]. All these expectations under the SDGs appear formidable and extremely challenging (Table 1); but, these goals are achievable provided that all actors and stakeholders at all levels are determined to achieve it.

However, we need to remember that a merely collaborative approach from scientists and/or academia is not enough to solve the challenging problems of society. To transform the world health-wise and achieve global sustainability by 2030, all actors and stakeholders, including all funding agencies and social scientists, must also play their roles effectively [3,4]. To attain these SDGs, everybody should be on the same page and there should be an efficient and effective cross talk on a regular basis among all actors and stakeholders (Figure 1).

Currently, it appears that the world community has recognized the importance of protecting our common home as it is our collective responsibility [5]. Along this line, if we make healthy living and the global sustainability our prime priority, then all public and private institutions around the globe need to align themselves with the SDGs. By bearing in mind the importance of healthy living and global sustainability for us and for our future generations, we need to act together.

In summary, achieving the SDGs is a huge and challenging task, but with intensive and well-organized result-oriented collaboration, effective cross talk among scientific communities, social scientists and people, policy makers and industry, and the strong determination of UN member states, we will define our success in making healthy living and the fate of future generations and global sustainability a reality.

## Figures and Tables

**Figure 1:**
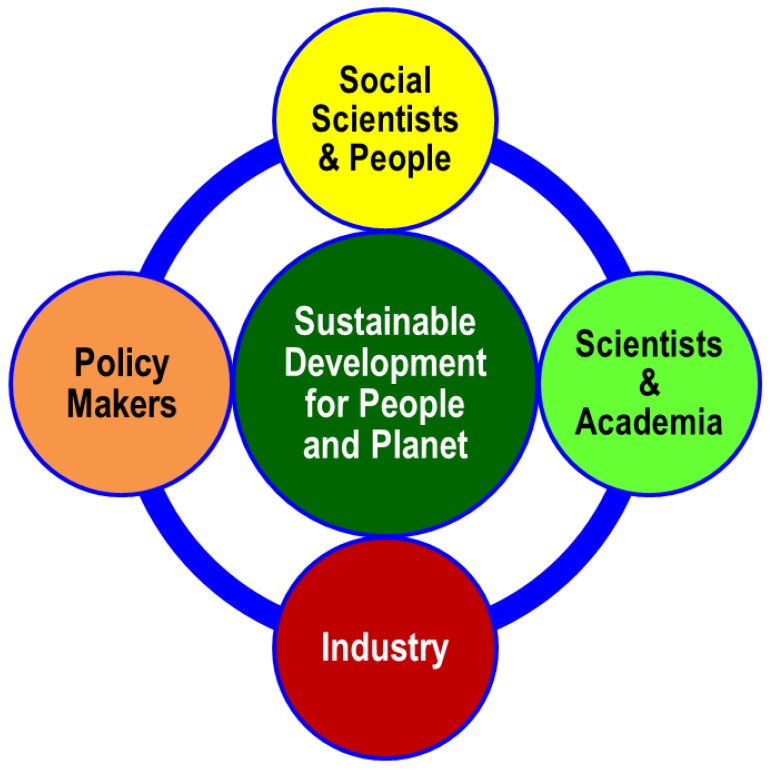
Stakeholders of global sustainability. All stakeholders should play their roles for sustainable global development.

**Table 1:** Promising and challenging Seventeen sustainable development goals adopted by United Nations (UN) [2].

No	Sustainable Development Goals	No	Sustainable Development Goals
***1***	End poverty in all its forms everywhere	***10***	Reduce inequality within and among countries
***2***	End hunger, achieve food security and improved nutrition and promote sustainable agriculture	***11***	Make cities and human settlements inclusive, safe, resilient and sustainable
***3***	Ensure healthy lives and promote well-being for all at all ages	***12***	Ensure sustainable consumption and production patterns
***4***	Ensure inclusive and equitable quality education and promote lifelong learning opportunities for all	***13***	Take urgent action to combat climate change and its impacts
***5***	Achieve gender equality and empower all women and girls	***14***	Conserve and sustainably use the oceans, seas and marine resources for sustainable development
***6***	Ensure availability and sustainable management of water and sanitation for all	***15***	Protect, restore and promote sustainable use of terrestrial ecosystems, sustainably manage forests, combat desertification, and halt and reverse land degradation and halt biodiversity loss
***7***	Ensure access to affordable, reliable, sustainable and modern energy for all	***16***	Promote peaceful and inclusive societies for sustainable development, provide access to justice for all and build effective, accountable and inclusive institutions at all levels
***8***	Promote sustained, inclusive and sustainable economic growth, full and productive employment and decent work for all	***17***	Strengthen the means of implementation and revitalize the global partnership for sustainable development
***9***	Build resilient infrastructure, promote inclusive and sustainable industrialization and foster innovation		
